# Biomechanical evaluation of seven fixation methods to treat pubic symphysis diastasis using finite element analysis

**DOI:** 10.1186/s13018-022-03078-5

**Published:** 2022-03-28

**Authors:** Yi-quan Zheng, Li-li Chen, Jia-zuo Shen, Bing Gao, Xiao-chuan Huang

**Affiliations:** 1 Department of Orthopaedics, Zhangzhou Affiliated Hospital of Fujian Medical University, No. 59, Sheng Li Road West, Xiangcheng District, Zhangzhou, 363000 Fujian China; 2 Department of Nursing, Zhangzhou Health Vocational College, Zhangzhou, China; 3 Department of Surgery, The People’s Hospital of Zhangzhou, Zhangzhou, China

**Keywords:** Pubic symphysis diastasis, Internal fixation, Subcutaneous fixation, Biomechanical evaluation

## Abstract

**Background:**

Pubic symphysis diastasis (PSD) hinders the connection between bilateral ischia and pubic bones, resulting in instability of the anterior pelvic ring. PSD exceeding 25 mm is considered disruptions of the symphyseal and unilateral/bilateral anterior sacroiliac ligaments and require surgical intervention. The correct choice of fixation devices is of great significance to treat PSD. This study aimed to evaluate the construct stability and implant performance of seven fixation methods to treat PSD using finite element analysis.

**Methods:**

The intact skeleton-ligament pelvic models were set as the control group. PSD models were simulated by removing relevant ligaments. To enhance the stability of the posterior pelvic ring, a cannulated screw was applied in the PSD models. Next, seven anterior fixation devices were installed on the PSD models according to standard surgical procedures, including single plates (single-Plate group), single plates with trans-symphyseal cross-screws (single-crsPlate group), dual plates (dual-Plate group), single cannulated screws, dual crossed cannulated screws (dual-canScrew group), subcutaneous plates (sub-Plate group), and subcutaneous pedicle screw-rod devices (sub-PedRod group). Compression and torsion were applied to all models. The construct stiffness, symphyseal relative micromotions, and von Mises stress performance were recorded and analyzed.

**Results:**

The construct stiffness decreased dramatically under PSD conditions. The dual-canScrew (154.3 ± 9.3 N/mm), sub-Plate (147.1 ± 10.2 N/mm), and sub-PedRod (133.8 ± 8.0 N/mm) groups showed better ability to restore intact stability than the other groups (*p* < 0.05). Regarding regional stability, only single-plate fixation provided unexpected regional stability with a diastasis of 2.1 ± 0.2 mm (*p* < 0.001) under a compressive load. Under a rotational load, the single-crsPlate group provided better regional angular stability (0.31° ± 0.03°, *p* < 0.001). Stress concentrations occurred in the single-Plate, sub-Plate, and sub-PedRod groups. The maximum von Mises stress was observed in the single-plate group (1112.1 ± 112.7 MPa, *p* < 0.001).

**Conclusion:**

The dual-canScrew fixation device offers ideal outcomes to maintain stability and prevent failure biomechanically. The single-crsPlate and dual-Plate methods effectively improved single-Plate device to enhance regional stability and disperse stresses. The subcutaneous fixation devices provided both anterior pelvic ring stability and pubic symphysis strength.

## Background

The pelvis is a complex geometrical structure comprising bilateral innominatums and sacrum connecting the trunk and the lower limbs [1, 2]. Disruption of the pelvic ring caused by high-energy trauma is a catastrophic and life-threatening injury [3, 4]. Pubic symphysis diastasis (PSD), with an incidence of 15–20% in pelvic fractures, is considered a Tile B1/anteroposterior compression (APC)-II lesion [5–8]. Pubic symphysis comprises bilateral pubic bones and intercalated fibrocartilaginous disks, which are mainly supported by four ligaments [1, 2]. A critical function of the pubic symphysis is to maintain the stability of the anterior pelvic ring. The pubic symphysis bears different types of forces under physiological conditions, including compression of the upper part and traction of the lower part when standing, shear and compression when single-leg standing, and compression when sitting [1]. Diastasis of the pubic symphysis indicates that the stability of the anterior pelvic ring is seriously damaged.

The anterior pelvic ring contributes approximately 40% of the structural stiffness and stability [9]. Correct treatment of PSD is critical to the quality of life of patients. The goal of treatment was to restore anatomical structure and stabilize the pelvic ring to improve the therapeutic effect and avoid complications [10]. Disruption of the pubic symphysis exceeding 25 mm is believed to be an absolute indication for operative intervention [5]. Several fixation strategies are used to enhance the stability of the anterior pelvic ring, such as external, internal, percutaneous, and subcutaneous fixation devices [6, 11–17]. Internal fixation of the anterior plate combined with posterior percutaneous cannulated screws is the preferred method to treat PSD, considering that 54–97% of anterior pelvic ring fractures are associated with posterior pelvic ring injuries [18–20].

A single plate may appear inadequate to fix disrupted pubic symphysis because it endures compression, traction, and shear forces under diverse physiological states [1]. Various novel fixation devices have been advocated to improve symphyseal stability. Dual plates and dual cannulated screws were used to strengthen the pubic symphysis region directly [10, 11]. Beder et al. evaluated the radiological and functional outcomes of symphyseal plates with trans-symphyseal cross-screws for the fixation of pelvic “open book” injuries [6]. The authors found that the novel device was a safe, efficient, and simple technique to treat PSD because of the biomechanical advantages of strengthened fixation to the inferior symphysis. Subcutaneous fixation systems with plates or pedicle screw-rod devices were introduced to fix anterior pelvic ring injuries [12, 15, 16]. The subcutaneous technique could provide sufficient biomechanical stability with fewer surgical complications, particularly for patients with hemodynamic instability or diabetes mellitus [21, 22]. Although novel fixation techniques have been applied clinically, the evaluation of biomechanical characteristics has not been reported. Therefore, this study aimed to evaluate the biomechanical characteristics of seven fixation methods to treat PSD using finite element analysis. The fixation methods included single plate, single plate with trans-symphyseal cross-screws, dual plates, single cannulated screw, dual crossed cannulated screws, subcutaneous plates, and subcutaneous pedicle screw-rod devices.

## Methods

### Finite element models and fixation devices

The imaging data were obtained from 0.65 mm thin-section computed tomography scans (Lightspeed VCT, GE, Fairfield, USA) of healthy volunteers (2 females and 3 males; 47.2 ± 9.7 years old; body mass index 20.4 ± 3.0). Finite element models of the pelvis were developed from imaging data using MIMICS 17.0 (Materialise, Belgium). The ligaments were added to the pelvis to create skeleton-ligament pelvic models in Abaqus 6.13 (3DS, Waltham, MA, USA), including superior pubic, arcuate pubic, sacrospinous, sacrotuberous, anterior sacroiliac, posterior sacroiliac, and interosseous sacroiliac ligaments. Nonlinear spring elements were used for all the ligaments. The connect type of springs was set as connect two points based on anatomical attachment points of ligaments. The material properties of the finite element models [12, 13] are shown in Table 1.

**Table 1:** The material properties of the finite element models

Materials		Elastic modulus (MPa)	Poisson ratio	Stiffness coefficient (N/m)	Number of springs
Skeleton	Cortical bone	17,000	0.3		
	Cancellous bone	129	0.2		
Ligaments	The superior pubic ligament			500	24
	The arcuate pubic ligament			500	24
	The sacrospinous ligament			1400	9
	The sacrotuberous ligament			1500	15
	The anterior and capsule sacroiliac ligament			700	27
	The posterior sacroiliac			1400	15
	The interosseous sacroiliac ligament			2800	8
Titanium implants		110,000	0.3		

**Fig. 1:**
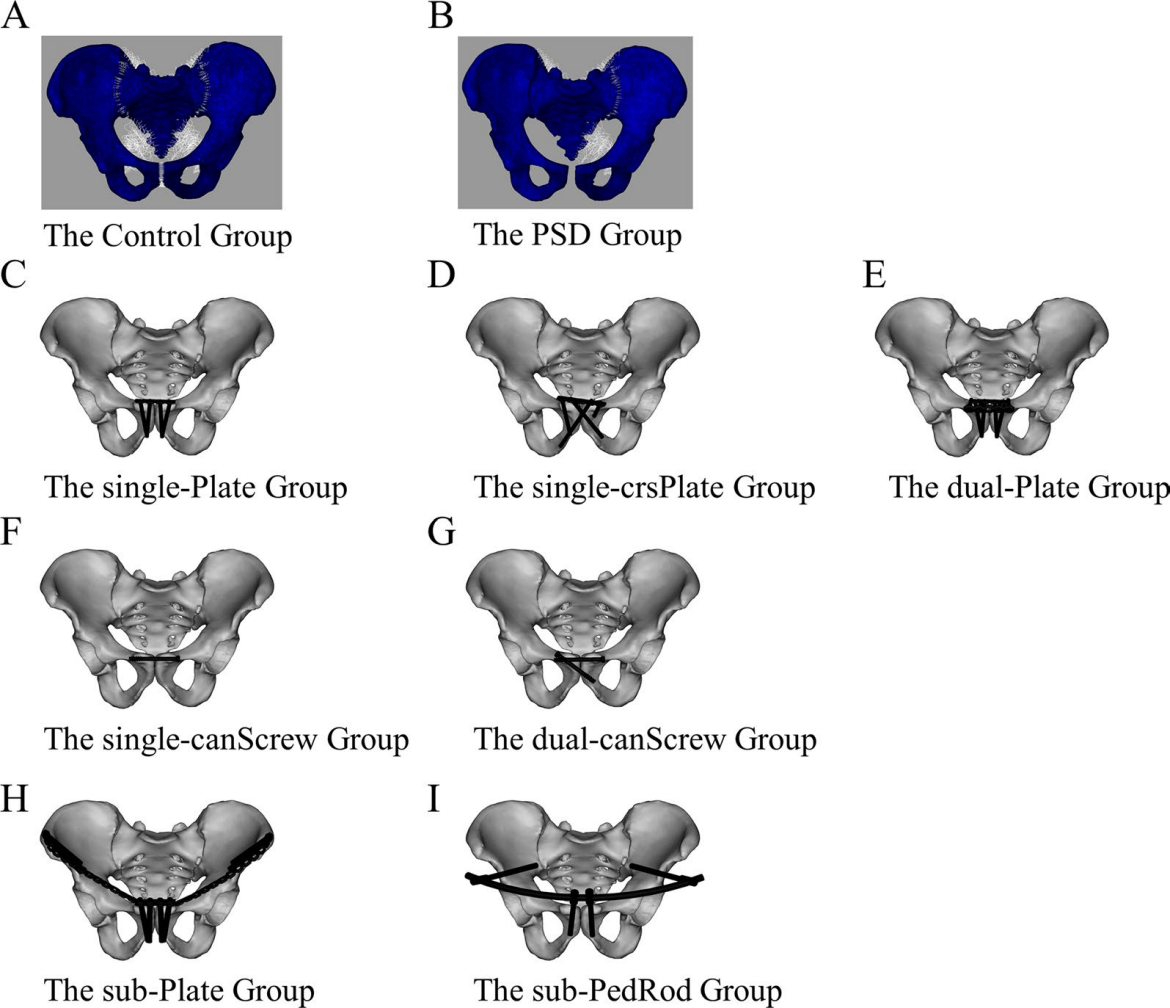
The intact skeleton-ligament pelvic models were set as the control group (**A**). The pubic symphysis diastasis group were simulated by removing the superior pubic, arcuate pubic, right sacrospinous, right sacrotuberous, and right anterior sacroiliac ligaments (**B**). Seven fixation devices were installed on pubic symphysis diastasis models, including single plate (**C**), single plate with trans-symphyseal cross-screws (**D**), dual plates (**E**), single cannulated screw (**F**), dual crossed cannulated screws (**G**), subcutaneous plates (**H**), and subcutaneous pedicle screw-rod devices (**I**)

The intact skeleton-ligament pelvic models were set as the control group (Fig. 1A). The PSD models (classical Tile B1/APC-II injury) were simulated by removing the superior pubic, arcuate pubic, right sacrospinous, right sacrotuberous, and right anterior sacroiliac ligaments (Fig. 1B). To enhance the stability of the posterior pelvic ring, a cannulated screw with a diameter of 7.3 mm was applied in the PSD model (PSD group). After posterior fixation, seven anterior fixation devices made of titanium alloy were installed on PSD models according to standard surgical procedures (Fig. 1C–I), including single plate (single-Plate group), single plate with trans-symphyseal cross-screws (single-crsPlate group), dual plates (dual-Plate group), single cannulated screw (single-canScrew group), dual crossed cannulated screws (dual-canScrew group), subcutaneous plates (sub-Plate group), and subcutaneous pedicle screw-rod (sub-PedRod group) devices. The reconstruction plates and the cannulated screws were obtained from dual-energy computed tomography scans. Four-hole, 5-hole, 4-hole and 5-hole reconstruction plates and two 17-hole reconstruction plates (DePuy Synthes, NJ, USA) used in the single-Plate, single-crsPlate, dual-plate, and sub-Plate groups, respectively. The length and diameter of the cannulated screws (DePuy Synthes, NJ, USA) were 60, 7.3 mm, and 60, 7.3 mm and 60, 6.5 mm in the single-canScrew group and dual-canScrew group, respectively. The pedicle screw-rod system was built by SolidWorks 2012 (3DS, Waltham, MA, USA) according to the manufacturer's specifications. The diameters of the pedicle screws and connecting rod (WeiGao Inc., WeiHai, China) were 8 and 8.5 mm in the sub-PedRod group, respectively.

The threads of all the screws were omitted to simplify the finite element models. Linear elastic isotropic material and deformable properties were assigned to the above elements. The rigid bond and surface-to-surface were set as the contact behavior of the screw-bone interface and plate-bone interface, respectively. The element type was set as quadratic tetrahedral elements (C3D10). The numbers of elements and nodes of the finite element models are shown in Table 2.

**Table 2:** The number of elements and nodes of the finite element models

Materials		Element	Node
Skeleton	Right innominatum bone	124,538	32,622
	Left innominatum bone	120,849	32,100
	Sacrum	155,023	43,441
Fixation devices	The sacroiliac screw	36,212	9186
	The single plate	39,769	9577
	The single plate with trans-symphyseal cross-screws	46,110	11,316
	The dual plates	71,431	17,486
	The single cannulated screw	25,304	6700
	The dual crossed cannulated screws	59,229	14,999
	The subcutaneous plates	189,779	45,638
	The subcutaneous pedicle screw-rod device	51,405	12,142

### Finite element analysis

**Fig. 2:**
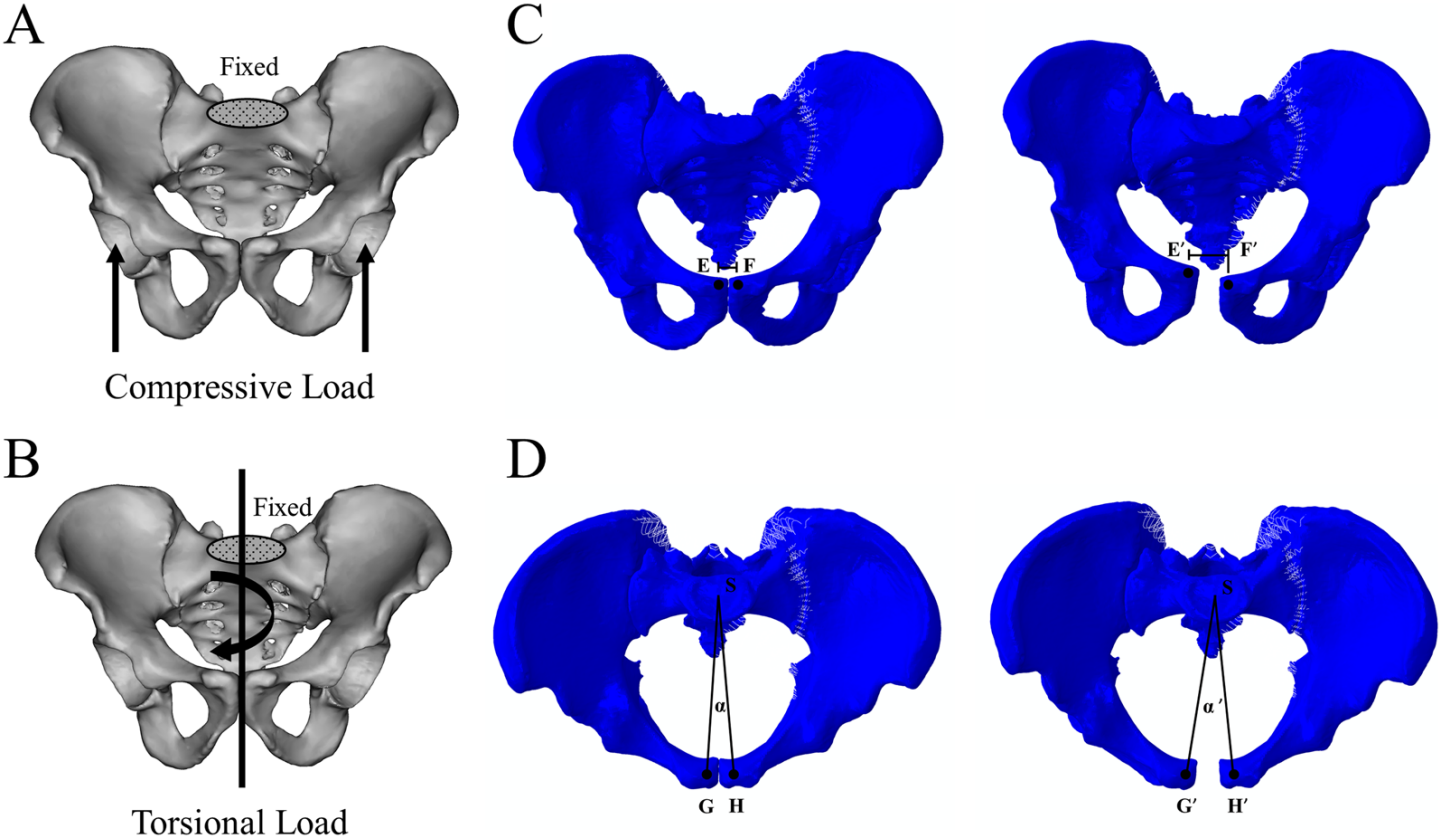
The load conditions of finite element analysis. The superior segment of S1 of the models was fixed. The compression (**A**) and torsion (**B**) were applied in all models. The relative displacement and rotational angle of the pubic symphysis were determined to display regional stability. The horizontal variation of distance between two reference (point **E** and **F**) points in bilateral pubic bones under compressive load was defined as regional compressive stability (**C**). The angular increment formed by two reference points (point **G** and **H**) in bilateral pubic bones and the center point of sacral endplate (point **S**) under torsional load was defined as regional torsional stability (**D**)

The finite element analysis was performed using Abaqus 6.13. The superior surface of the sacrum was fixed in all the finite element models. The once compression and torsion were applied to all models for static analysis. For compression, load of 550 N was distributed onto the acetabulum (Fig. 2A) to ensure that the force required to simulate maximum pubic symphysis mobility as load of 333 N in the vertical direction and load of 136 N in the sagittal direction [23, 24]. For torsion, a 10 Nm torque was applied to pelvic models (Fig. 2B).

Evaluation of biomechanical characteristics in the immediate postoperative period was performed from three aspects. The intact stability of the pelvis was assessed by construct stiffness. The construct stiffness was determined from the slope of the intact pelvic force–displacement graphs. The relative displacement and rotational angle of the pubic symphysis were determined to display regional stability. The horizontal variation of distance between two reference points in bilateral pubic bones under compressive load was defined as regional compressive stability (Fig. 2C). The angular increment formed by two reference points in bilateral pubic bones and the center point of sacral endplate under torsional load was defined as regional torsional stability (Fig. 2D). The von Mises stress distribution and maximum stresses on fixation devices were investigated to understand the performance of implants.

### Statistical analysis

Statistical analyses were calculated using SPSS software (version 19.0; SPSS Inc., Chicago, IL). All the data were analyzed to determine the normal distribution using the Kolmogorov–Smirnov test. One-way analysis of variance was performed to compare the means among all the groups. Independent-samples *t*-test was used to compare the means between two groups. A value of *p* < 0.05 was considered statistically significant.

## Results

### Validation of the finite element model

**Fig. 3:**
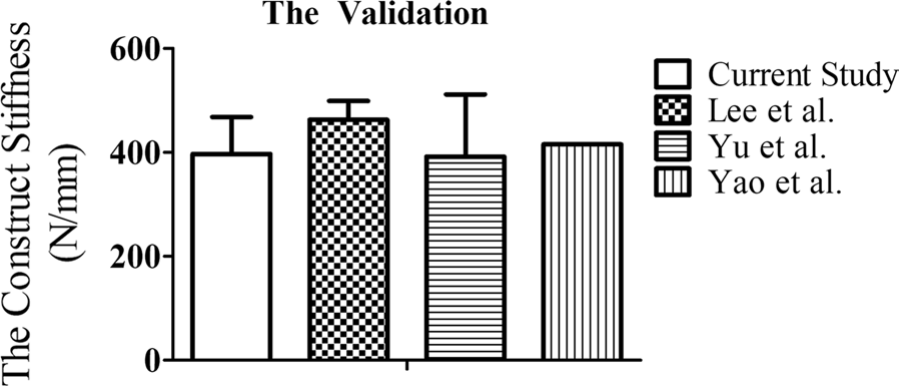
The validation of the finite element model. The current study showed close agreement with previous studies by Lee et al. [25], Yu et al. [26], and Yao et al. [11]. The results indicated that the intact pelvic model was validated for further analysis

The comparison between the present study and pervious research regarding the construct stiffness of intact specimen is shown in Fig. 3. The current study showed close agreement with previous studies by Lee et al. [25], Yu et al. [26], and Yao et al. [11]. The results indicated that the intact pelvic model was validated for further analysis.

### Intact stability of the pelvis

**Fig. 4:**
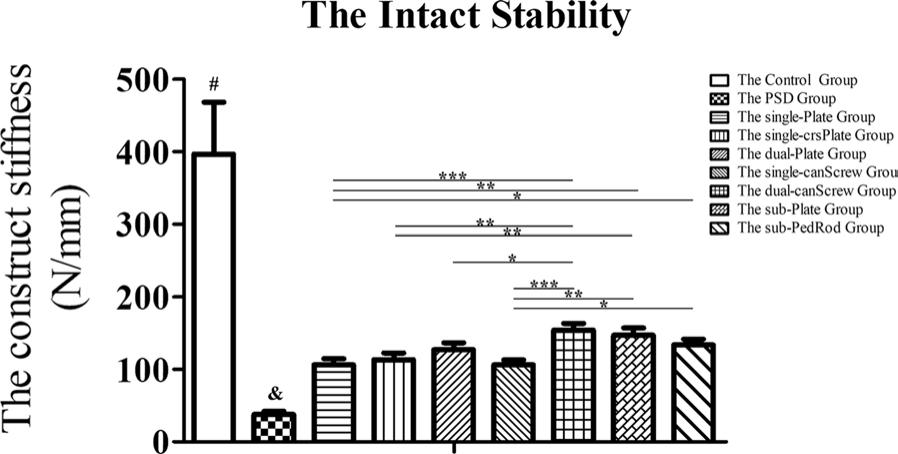
The results of pelvic construct stiffnesses under compression loading. ^#^ means *p* < 0.05 between the control group and other groups; ^&^ means *p* < 0.05 between the PSD group and other groups; ^*^ means *p* < 0.05; ^**^ means *p* < 0.01; and ^***^ means *p* < 0.001

The construct stiffnesses of the pelvis under compressive load are displayed in Fig. 4. The construct stiffness decreased dramatically after removing the superior pubic, arcuate pubic, right sacrospinous, right sacrotuberous, and right anterior sacroiliac ligaments. All fixation devices did not achieve the intact stability of the control group. Generally, the dual-canScrew (154.3 ± 9.3 N/mm), sub-Plate (147.1 ± 10.2 N/mm), and sub-PedRod (133.8 ± 8.0 N/mm) groups showed better construct stiffness than the single-Plate (106.3 ± 8.3 N/mm), single-crsPlate (113.4 ± 9.1 N/mm), dual-Plate (127.3 ± 9.6 N/mm), and single-canScrew (106.5 ± 6.7 N/mm) groups (*p* < 0.05).

### Regional stability of pubic symphysis

**Fig. 5:**
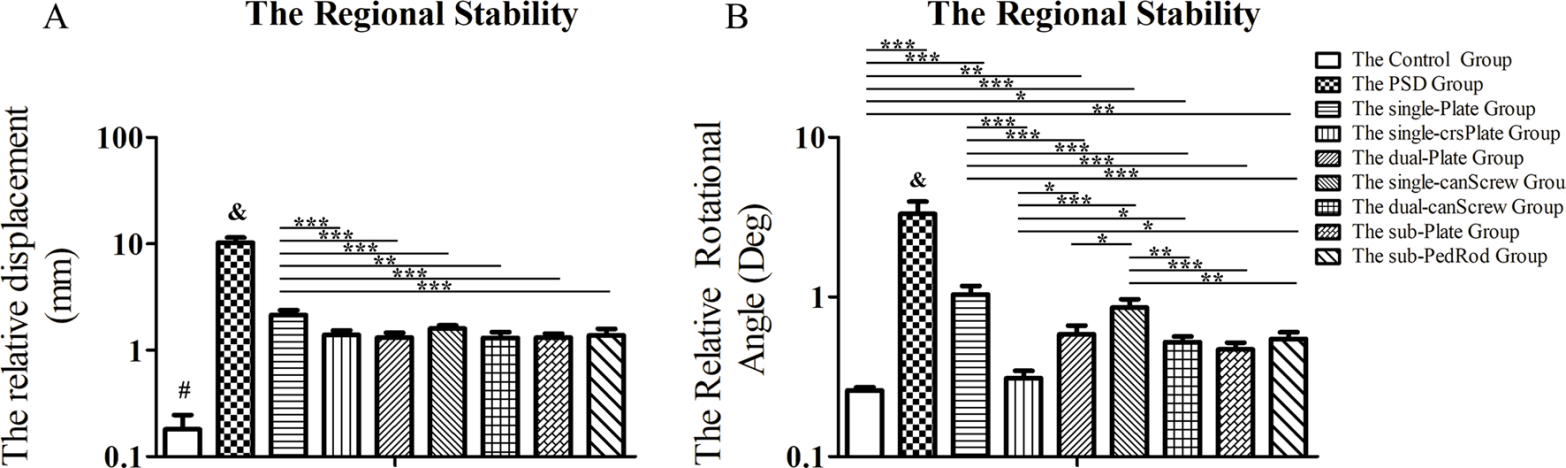
The outcomes of regional stability of the pubic symphysis. ^#^ means *p* < 0.05 between the control group and other groups; ^&^ means *p* < 0.05 between the PSD group and other groups; ^*^ means *p* < 0.05; ^**^ means *p* < 0.01; and ^***^ means *p* < 0.001

The regional stability of the pubic symphysis is shown in Fig. 5. The displacement increments of the symphyseal region under compressive load were 0.2 ± 0.1 mm under physiological conditions, 28.4 ± 2.0 mm under PSD conditions, and 10.3 ± 1.2 mm under PSD after posterior iliosacral screw fixation. Diastasis of 28.4 ± 2.0 mm reached the surgical indication (> 25 mm). Under compressive load, most of the fixation devices provided preferred regional stability with a diastasis of less than 1.6 mm, except single-Plate fixation with diastasis of 2.1 ± 0.2 mm (*p* < 0.001). Under a torsional load, the single-crsPlate (0.31° ± 0.03°) group provided better regional angular stability than the other fixation methods (*p* < 0.001), while the single-Plate (1.04° ± 0.14°) and single-canScrew (0.86° ± 0.11°) groups showed unsatisfactory results.

### The von Mises stress of fixation devices

**Fig. 6:**
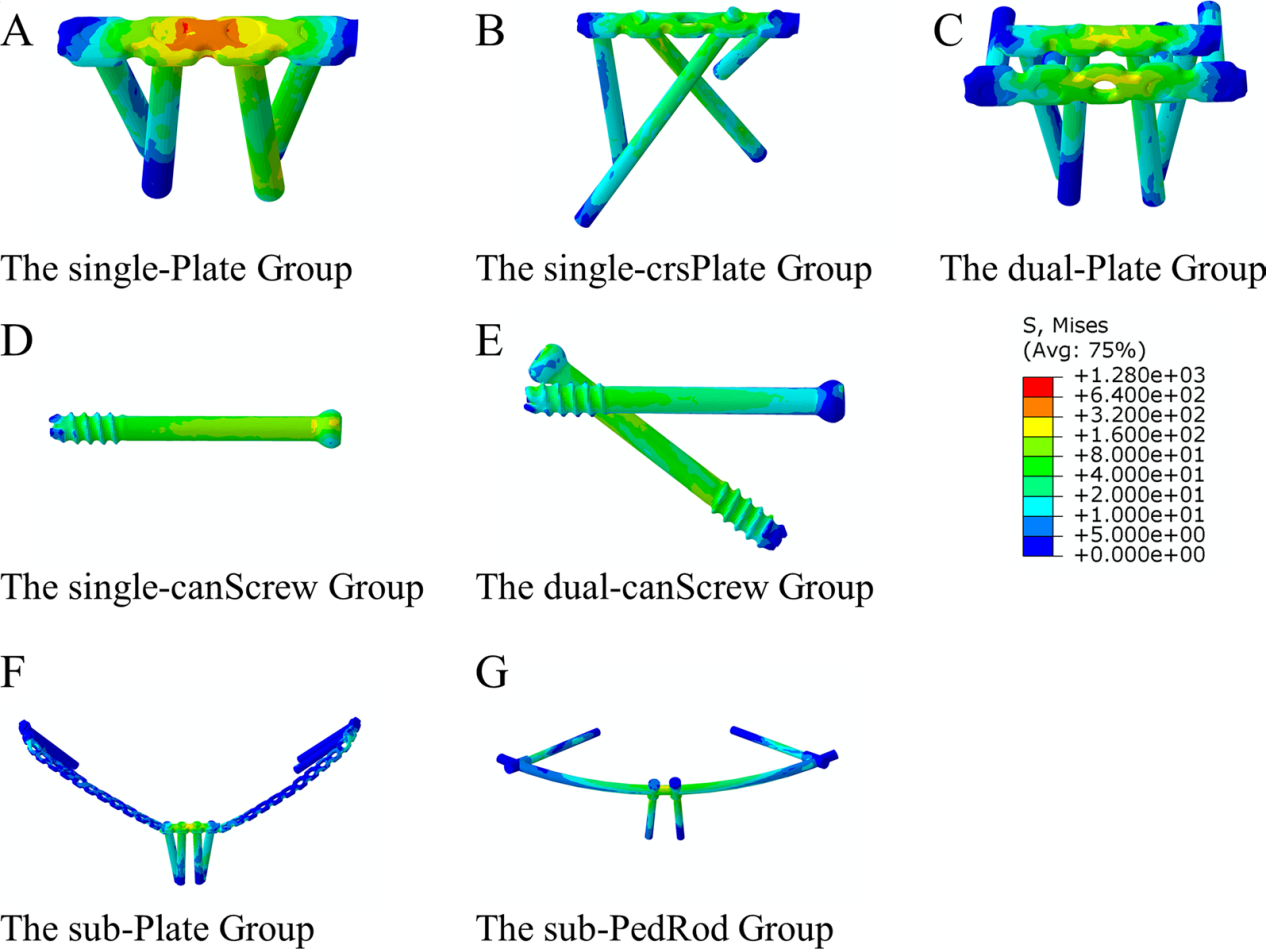
The von Mises stress distributions on different fixation devices under compressive load condition

**Fig. 7:**
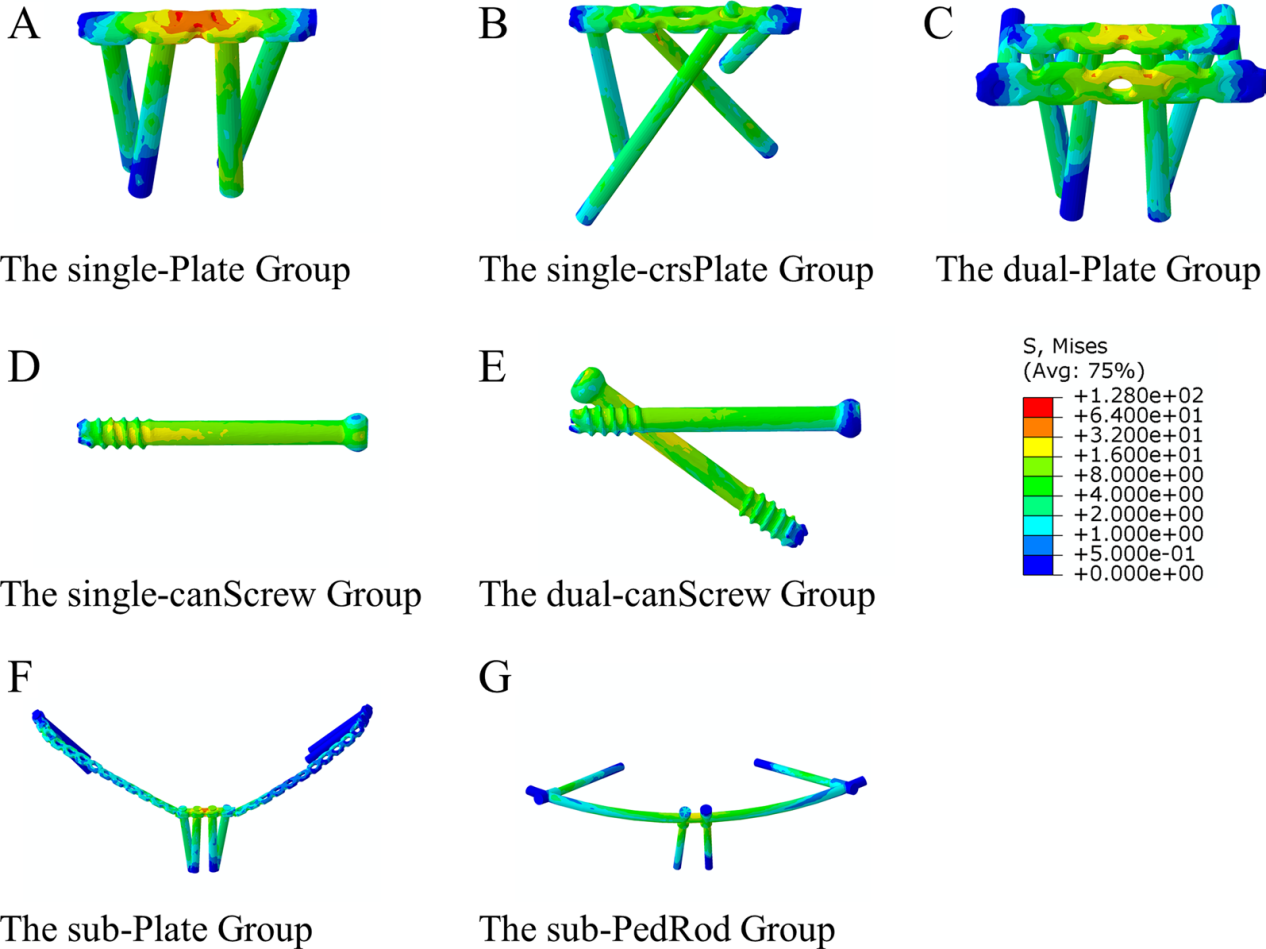
The von Mises stress distributions on different fixation devices under torsional load condition

The von Mises stress distributions on different fixation devices under compressive and torsional loads are displayed in Figs. 6 and 7. The von Mises stress was concentrated in the area where the implant crossed the pubic symphysis in the single-Plate, sub-Plate, and sub-PedRod groups. In the dual-Plate group, the concentrated stress on the superjacent plate was dispersed by an additional plate. Stress concentration was inconspicuous in the dual-canScrew, single-canScrew, and single-crsPlate groups. The features of stress distribution on implants were similar under compressive and torsional loads.

**Fig. 8:**
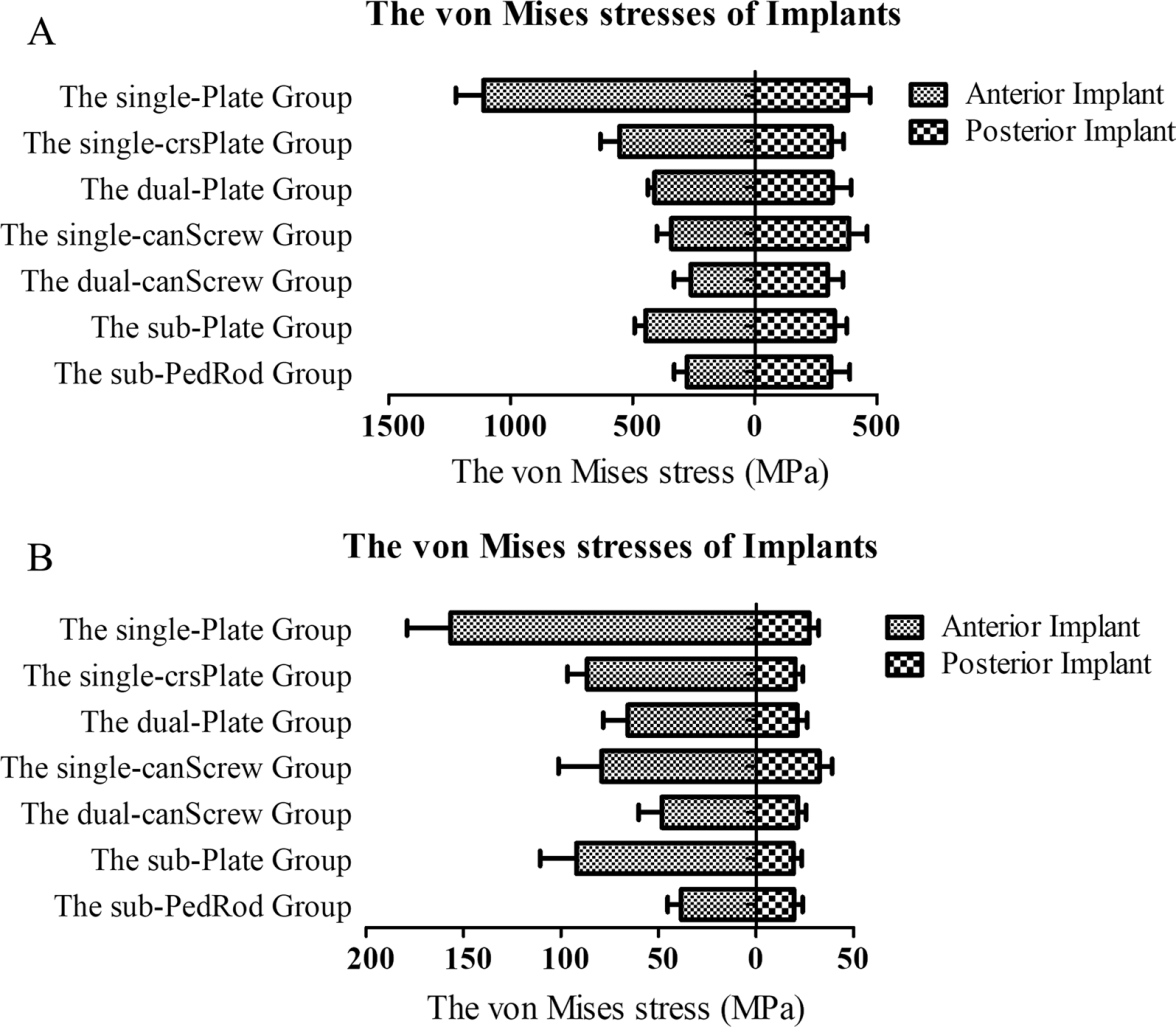
The maximum von Mises stress on anterior and posterior implants

The maximum von Mises stress on implants is displayed in Fig. 8. The maximum von Mises stress occurred in the single-Plate group under compressive (1112.1 ± 112.7 MPa) and torsional (156.7 ± 22.3 MPa) load conditions (*p* < 0.001). The implants in the dual-canScrew and sub-PedRod groups endured less stress than those in the other groups (*p* < 0.05). The posterior iliosacral screw endured larger von Mises stresses in the single-Plate and single-canScrew groups under compressive and torsional loads (*p* = 0.042 and < 0.001); however, the values of von Mises stresses were small.

## Discussion

The pubic symphysis provides a connection between bilateral ischia and pubic bones with physiological micromotion under functional loads [1, 2, 27]. Surgical intervention is recognized when PSD exceeds 25 mm, which means the disruptions of symphyseal and anterior sacroiliac ligaments [5, 27]. Several rigid fixation devices have been used to treat PSD. The present study aimed to evaluate the biomechanical characteristics of seven fixation methods to treat PSD.

Fixation with a single anterior plate and posterior iliosacral screw is recommended as a classic therapeutic strategy for PSD [19, 20]. However, the failure rates of pubic plates from 12 to 75% have attracted extensive attention [6]. In our study, we found that the maximum von Mises stress of 1112.1 ± 112.7 MPa occurred in the single-plate group under compressive loading. The maximum stress greater than 880 MPa (the yield strength of titanium alloy) implies that the implant will likely fail. Additionally, the stress concentration appeared at the middle part of a single plate where the plate crossed the pubic symphysis. The unsatisfactory results may explain the high failure rate of a single plate, which should not be considered as the first choice for PSD treatment. The dual planar fixation system with superior and anterior symphyseal plates was advocated to enhance regional stability and disperse stresses. In our results, the increment of symphyseal displacement decreased from 2.1 ± 0.2 mm in the single-Plate group to 1.3 ± 0.1 mm in the dual-Plate group. Evidently, the concentrated stress on the superior plate was dispersed by the anterior plate. A single plate with trans-symphyseal cross-screws was introduced to treat pelvic “open book” injuries. Beder et al. evaluated the radiological and functional results and found that a single-crsPlate fixation device was a safe, efficient, and simple technique to treat PSD [6]. The authors believed that the trans-symphyseal cross-screws provided biomechanical stability, particularly for strengthened fixation to the inferior symphysis. Our study further proved the effect of a single-crsPlate fixation device on enhanced symphyseal regional stability. The single-crsPlate group provided positive regional anti-rotational ability (0.31° ± 0.03°) and anti-compressive ability (1.4 ± 0.1 mm). Additionally, the satisfactory symphyseal stability may be obtained by cross fixation and the strengthened holding force of long trans-symphyseal cross-screws. Generally, the single-crsPlate and dual-Plate methods were effective improvements to the single-Plate device to enhance regional stability and dispersing stresses.

The minimally invasive surgical (MIS) technique is the ideal method to minimize surgical complications, such as blood loss, deep surgical site infection, and iatrogenic vascular and nerve injuries. Anterior percutaneous cannulated screws are the most frequently used MIS method to treat PSD [11, 13]. Yu et al. reported the biomechanical properties and clinical outcomes regarding percutaneous cannulated screw and plate devices to treat PSD [13]. The authors found that the two fixation methods displayed similar biomechanical characteristics to treat Tile B1 injuries. Additionally, the percutaneous cannulated screw technique exhibited advantages regarding fewer surgical complications, including less blood loss, a shorter operative time, and fewer scars. Yao and colleagues compared the biomechanical characteristics using different fixation devices to stabilize PSD [11]. They declared that dual cannulated screws provided higher stiffness than plate fixation devices. Furthermore, the parallel and crossed dual cannulated screws showed the preferable fixation effect under dual-leg and single-leg stances. In our study, the dual-canScrew device offered the ideal overall performance with better intact and regional stability. The preferable results of the dual-canScrew group benefited from enhanced symphyseal stability with dual screw fixation and fair anti-rotation ability with a crossed arrangement. The inferior part fixation of the pubic symphysis could resist traction forces directly. Furthermore, the von Mises stresses were evenly distributed on the cannulated screws, helping to avoid implant failure. In the current study, different anterior fixation methods had a slight impact on the posterior device. The maximum von Mises stress on the posterior cannulated screw was higher in the single-Plate and single-canScrew groups; however, the stresses in all the groups were lower than the yield strength of titanium alloy (880 MPa [28]). The result may suggest that the failure probability of the posterior cannulated screw is low by finite element analysis. The clinical influence of different anterior fixation methods on posterior cannulated screw needs to be further explored. Generally, the dual-canScrew fixation device may be an ideal choice for definitive fixation to maintain stability of pelvic ring and prevent implant failure biomechanically.

Recently, another novel MIS technology of subcutaneous fixation was frequently mentioned. The subcutaneous fixation was introduced to treat anterior pelvic injuries as a temporary or definitive fixation, particularly for patients with hemodynamic instability or diabetes mellitus [12, 15, 16]. The advantage of subcutaneous devices is to achieve a balance among a short operation time, fewer surgical complications, and a reliable fixation strength. Wang et al. used a modified subcutaneous pedicle screw–rod fixation method to stabilize PSD [16]. Through the retrospective analysis of clinical and radiological outcomes in 29 patients, the authors declared that this modified fixator was an alternative method to treat PSD with early weight-bearing. Jordan and colleagues compared the biomechanical properties between anterior subcutaneous and external fixation devices to stabilize disrupted pelvic rings [15]. They found no significant differences between different methods. A potential risk was reported: the curved connecting rod was easily displaced within the polyaxial screw head and may harm the surrounding anatomical structures. In our study, the sub-Plate and sub-PedRod groups presented better intact pelvic stability than the other fixation groups. This result may be explained by the subcutaneous fixator strengthening the anterior pelvic ring stability by fixing the bilateral ilia and middle pubic symphysis. However, subcutaneous devices failed to provide stronger regional stability than dual-Plate and dual-canScrew fixators with a compressive load and single-crsPlate and dual-canScrew fixators with a rotational load. This finding suggests that surgeons should fully consider both anterior pelvic ring and symphyseal injuries when choosing the preferred fixation method. In the subcutaneous fixation groups, the stress concentration was also displayed on the part of fixators where it strides over the pubic symphysis. The connection plates/rods between the pubic symphysis and ilium dispersed the stresses and may prevent implant failure. Overall, the subcutaneous plates and pedicle screw-rod devices offered both anterior pelvic ring stability and pubic symphysis strength simultaneously from a biomechanical view.

The major limitation of the present biomechanical study was that the pelvic models were simulated based on the skeleton-ligament system while ignoring the influence of soft tissues. The current modeling strategy is a common method when performing finite element studies. Additionally, the compressive and torsional loads were simulated in our study, mismatching the complexity of physiological conditions. However, the gold standard method to perform biomechanical analysis regarding PSD has not been established, especially for simulating gait analysis. The current study aimed to analyze the biomechanical outcomes of different fixators under compressive and torsional loading conditions. The present study provided biomechanical clues for our further mechanical test, fatigue test, and clinical investigations. Furthermore, we made a classical PSD (Tile B1/APC-II lesion) in current study, which was not enough to represent all injury cases. The gap between the experimental conditions and the clinical situations (different types of ligament injuries) weakened the clinical correlation of our study. This limitation brought difficulties to improve the clinical application through the experimental results. The results only provided references for clinical application by finite element analysis. The surgeons should make reasonable treatment plans according to the specific situation in clinical practice, such as combined with other fractures, different degree of ligament injuries, and expert experience. Finally, our study analyzed immediate postoperative biomechanical stability without considering long-term outcomes. Because of these limitations, the results of the present study must be carefully considered before being used as a clinical reference.

## Conclusion

The present study evaluated the biomechanical characteristics of seven fixation methods to treat PSD using finite element analysis. Generally, the dual-canScrew fixation device may be an ideal choice to maintain stability and prevent implant failure. The single-crsPlate and dual-Plate methods effectively improved single-Plate device to enhance regional stability and disperse stresses. The subcutaneous fixation devices offered both anterior pelvic ring stability and pubic symphysis strength.

## Data Availability

The datasets used and/or analyzed during the current study are available from the corresponding author on reasonable request.

## Abbreviations

APC-II: Anteroposterior compression-II
Dual-canScrew: Dual crossed cannulated screws
Dual-Plate: Dual plates
MIS: Minimally invasive surgery
PSD: Pubic symphysis diastasis
Single-canScrew: Single cannulated screw
Single-crsPlate: Single plate with trans-symphyseal cross-screws
Single-Plate: Single plate
Sub-PedRod: Subcutaneous pedicle screw-rod
Sub-Plate: Subcutaneous plates
